# Explainable machine learning and physics-constrained optimization of chalcogen catalysts for sustainable hydrogen production

**DOI:** 10.1016/j.isci.2026.117213

**Published:** 2026-08-24

**Authors:** Du Nguyen, M. Olga Guerrero-Pérez, Enrique Rodríguez-Castellón, Minh Chuong Nguyen, Hadiyanto Hadiyanto, Van Hoc Le, Van Huong Dong, Xuan Phuong Nguyen, Anh Tuan Hoang

**Affiliations:** 1 Institute of Engineering, HUTECH University, Ho Chi Minh City, Vietnam; 2 Department of Chemical Engineering, School of Industrial Engineering, University of Málaga, 29071 Málaga, Spain; 3 Department of Inorganic Chemistry, Faculty of Science, Interuniversitary Institute for Research in Biorefineries I3B, University of Malaga, 29071 Málaga, Spain; 4 Institute of Research and Development, Duy Tan University, Da Nang, Vietnam; 5 School of Engineering and Technology, Duy Tan University, Da Nang, Vietnam; 6 Chemical Engineering Department, Faculty of Engineering, Diponegoro University, Semarang, Indonesia; 7 Center of Biomass and Renewable Energy (CBIORE), Diponegoro University, Semarang, Indonesia; 8 Faculty of Engineering, Dong Nai Technology University, Dong Nai, Vietnam; 9 PATET Research Group, University of Transport Ho Chi Minh City, Ho Chi Minh City, VietNam; 10 Graduate School of Energy and Environment, Korea University, 145 Anam-ro, Seongbuk-gu, Seoul 02841, South Korea

**Keywords:** band gap prediction, chalcogen electrocatalyst, hydrogen production, physics-constrained optimization, trust-region Bayesian optimization, SHAP analysis

## Abstract

The accelerating demand for clean energy has intensified research on sustainable hydrogen production, where efficient chalcogen catalyst design remains a major challenge. Most of the existing studies are, however, only concerned with predictive modeling, neglecting physical feasibility and consistency in the optimization process. To overcome this, this study proposes an integrated machine learning and optimization framework, physics-constrained optimization (PCO) and trust-region Bayesian optimization (TRBO), for engineering of chalcogen-based electrocatalysts. The proposed framework takes into account domain-specific physical band gap constraints, such as physically stable formation energies and realistic density bounds, to guarantee physically meaningful and practically feasible solutions. The XGBoost model trained with engineered Magpie descriptors has demonstrated excellent predictive accuracy with R^2^ of 0.961 along with low prediction error. PCO also showed better results with the highest band gap of 2.406 eV as compared with the 2.276 eV of TRBO. SHapley Additive exPlanations (SHAP) analysis also revealed the most influential descriptors that govern the behavior of the band gap, enhancing the interpretability of the model. In summary, the proposed framework offers a physically consistent, reliable, and scalable route for the discovery of new catalysts and sustainable hydrogen production.

## Highlights

• Sustainable hydrogen production could be boosted by chalcogen electrocatalysts
• Integrated machine learning and optimization framework for band gap engineering
• XGBoost achieves an R^2^ of 0.961 (train) and 0.956 (test) with low prediction error
• PCO and TRBO optimize the band gap to 2.406 and 2.276 eV, respectively

## Graphical abstract

**Figure:**
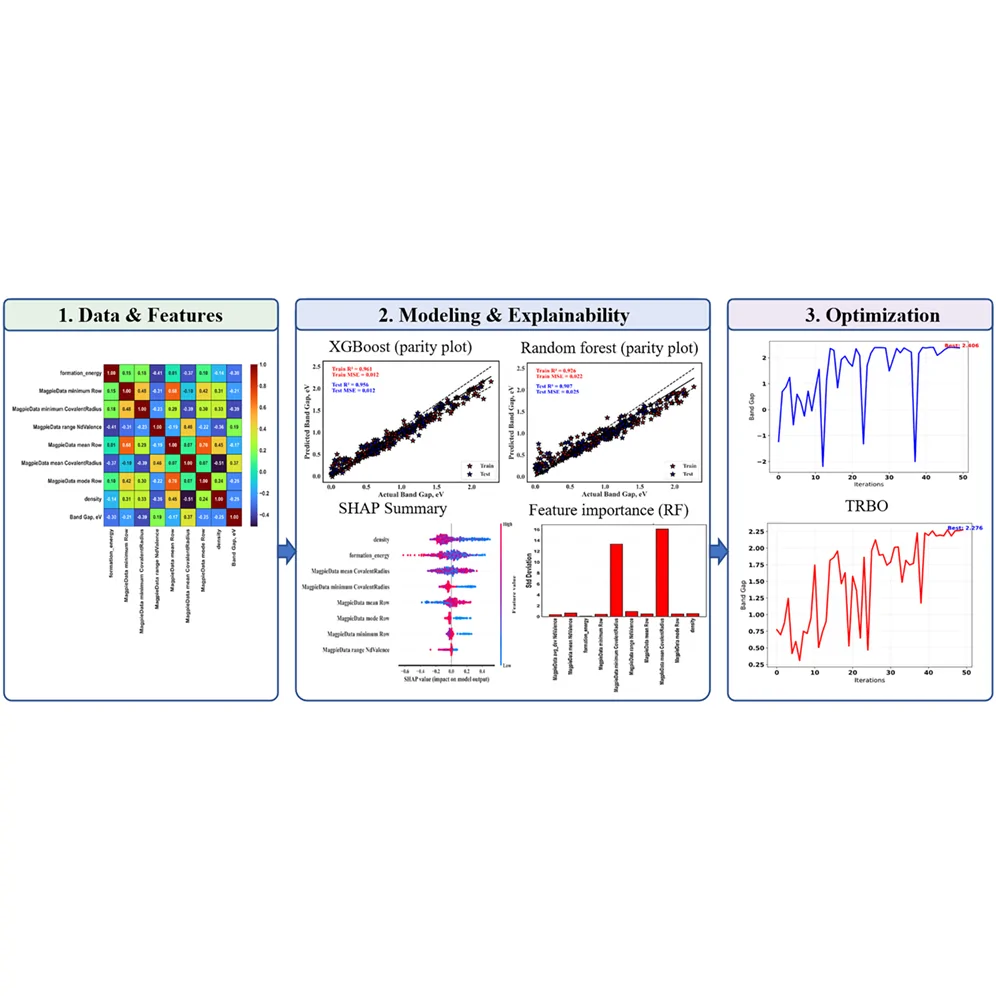

## Teaser

chemistry; catalysis; electrochemistry; applied sciences

## Introduction

For many decades, fossil fuels have been known as the most prevalent energy source, but it has contributed significantly to greenhouse gas emissions and climate change. In addition, the global energy demand in the world is ever-increasing due to the increase in population and the development of industry, resulting in the depletion of fossil energy and fuel sources.^1^^,^^2^ Facing the rapid growth in energy demand and the negative impacts of fossil fuel use, looking for sustainable and low-carbon energy sources has become a priority.^3^^,^^4^ Indeed, recent research points out that the shift to sustainable and low-carbon energy systems is necessary to reach net-zero goals by mid-century.^5^^,^^6^ In this regard, hydrogen has become a clean and multi-purpose energy carrier because it has a high energy density and zero carbon emissions at the point of use.^7^^,^^8^ Among various hydrogen production pathways, green hydrogen, produced through water electrolysis using renewable energy, is one of the most sustainable pathways.^9^^,^^10^^,^^11^ Nevertheless, to scale up the process, efficient, stable, and cost-effective electrocatalysts are needed to catalyze the hydrogen evolution reaction (HER) at low overpotentials and high current densities.^12^^,^^13^ HER is a basic electrochemical reaction that takes place at the cathode during water splitting, which follows well-known processes, i.e., the Volmer-Heyrovsky and Volmer-Tafel pathways, which are determined by the reaction environment and the catalyst surface. These processes entail electron transfers that are proton-coupled, followed by hydrogen recombination. However, the reaction kinetics are very sensitive to the hydrogen adsorption free energy (ΔG_H∗), which ideally should be close to the thermoneutrality to achieve optimal catalytic activity.^14^^,^^15^

**Figure 1:**
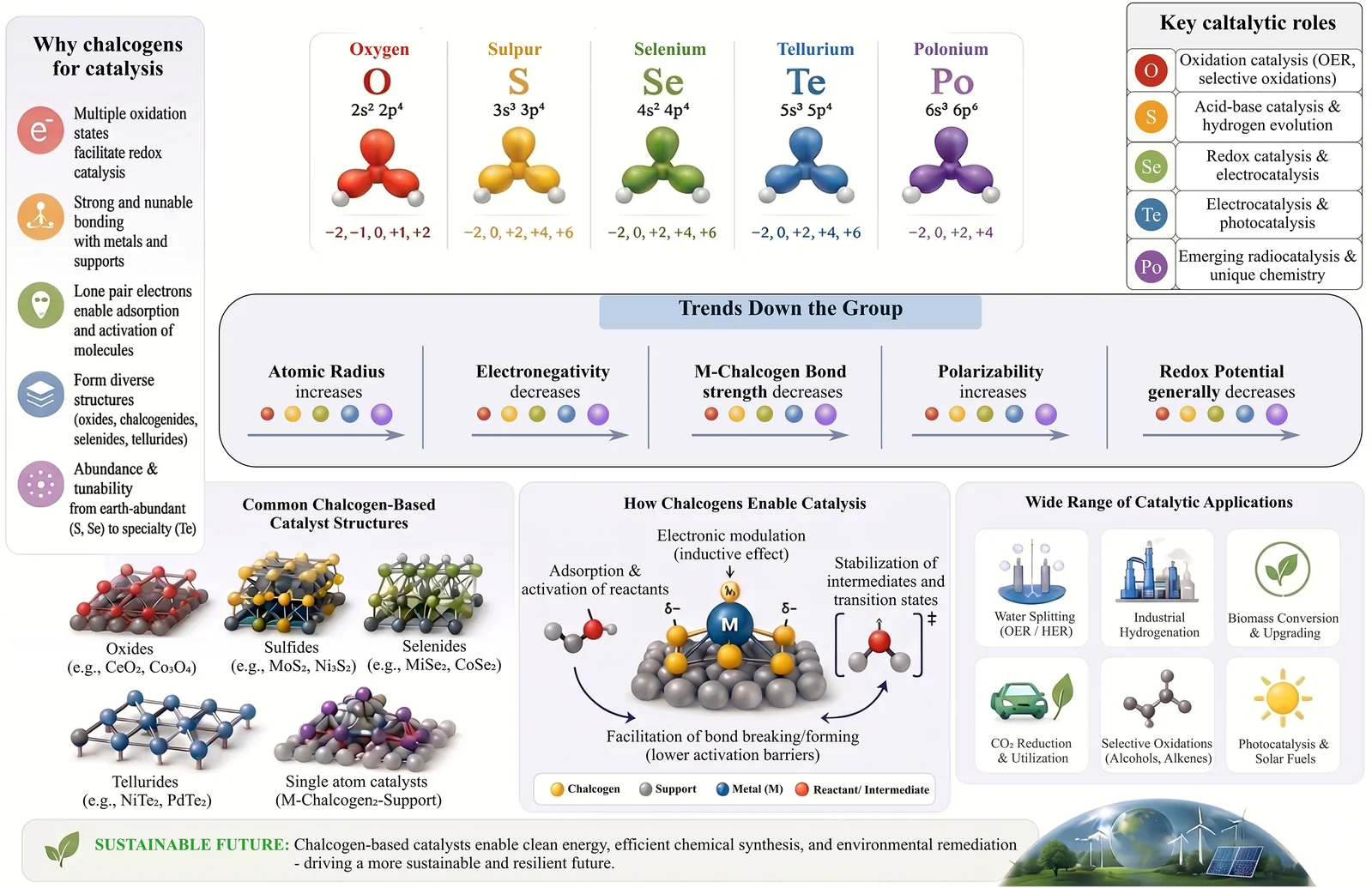
Chalcogen elements and the critical properties of chalcogen-based catalysts (the Lv element was excluded because it is an artificially synthesized radioactive element)

Platinum (Pt)-based catalysts are commonly considered the standard HER catalysts, since they have near-zero ΔG_H∗ and rapid reaction kinetics. Indeed, low Tafel slopes (∼30 mV/dec) have been consistently reported in both experimental and theoretical studies for Pt surfaces, suggesting that these surfaces are catalytically active. Nevertheless, Pt is very scarce and expensive, which greatly limits its widespread use and encourages the creation of other catalysts.^16^ Recent studies have been directed at decreasing the use of noble metals by developing bimetallic and hybrid catalysts. One example is platinum-based alloys, such as Pt/Ru and Pt/Ni, that enhance catalytic activity by electronic (ligand) and geometric (strain) effects, altering the d-band center and maximizing the strength of hydrogen adsorption. In addition to noble metal systems, there has been considerable advancement in the development of non-precious electrocatalysts. Among inexpensive co-catalysts such as carbides, phosphides, borides, MXenes, and hydroxides,^17^^,^^18^^,^^19^ chalcogen-based catalysts have emerged as highly promising candidates for photocatalytic hydrogen evolution owing to their earth abundance, affordability, diverse compositions, rich structural diversity, and readily adjustable atomic and electronic structures.^20^^,^^21^ The critical properties of chalcogen elements and the catalytic application of chalcogen elements are presented in Figure 1.

Originating from chalcogen elements, there are some main catalyst types used for hydrogen production, such as transition metal dichalcogenide (TMD) (including MoS_2_ and WSe_2_, which possess binding energies similar to expensive Pt-based catalysts but are much cheaper than Pt and have high durability in acid)^22^; metal sulfides and selenides (normally including CdS, ZnS, Cu_2_S, and CuSe, which have excellent band-gap alignment for promoting water splitting)^23^; and ternary chalcogenides (I-III-VI_2_ with VI of S, Se, or Te, and I/III as metal).^24^ Recent surveys highlight that next-generation catalysts are of research interest to attain industrial-level durability and high activity.^25^^,^^26^ Among these, two-dimensional (2D)-TMDs are emerging catalysts that have received significant interest because of their distinctive electronic characteristics, large surface area, and controllable catalytic activity. TMDs have been shown to increase catalyst stability and decrease the loading of noble metals required.^27^^,^^28^ Nevertheless, the basal planes of pristine TMDs are usually inert, which restricts their overall activity.^29^^,^^30^ To address this drawback, several methods have been developed, including defect engineering, strain modulation, and phase transformation. Defect engineering, especially the formation of chalcogen vacancies, is very important for improving HER performance.^31^^,^^32^^,^^33^ Vacancies in MoS_2_, such as sulfur, create unsaturated metal sites, which greatly enhance hydrogen adsorption and lower ΔG_H∗. Computational analyses have revealed that these defect sites can reduce reaction barriers and increase catalytic turnover frequencies. Furthermore, layered chalcogen-based catalysts such as MoS_2_, MoSe_2_, and WS_2_ have edge active sites that promote HER. Pt systems supported on MoS_2_ exhibit enhanced nanoparticle dispersion and agglomeration resistance, and thus, catalytic activity during extended operation.^34^^,^^35^ Similarly, strain engineering has been used to modify the electronic structure of TMDs. Tensile or compressive strain changes the d-band structure and maximizes contact between the catalyst surface and hydrogen intermediates, enhancing catalytic efficiency.^36^^,^^37^^,^^38^ Another promising approach to improving TMD performance is phase engineering. Although the 2H semiconductor phase is the most common, the metallic 1T7 phase has higher electrical conductivity and is more catalytically active. Recent studies have been keen on stabilizing this metastable phase. It has been shown that 1T-phase MoS_2_ can attain much lower overpotentials and faster charge transfer kinetics than its 2H counterpart. These results indicate the significance of tuning the electronic structure in catalyst design.^39^^,^^40^ Besides traditional TMDs, more sophisticated designs like Janus monolayers and alloyed systems have been considered. Asymmetric chalcogen layers in Janus TMDs generate intrinsic dipole moments that increase charge separation and adsorption capabilities. As an example, WSeTe Janus monolayers have demonstrated positive reaction energetics and lower energy barriers for HER, making them promising high-performance catalysts.^41^ Likewise, electronic properties, defect densities, and catalytic activity can be fine-tuned by alloying with various chalcogen elements.^39^^,^^40^^,^^42^ Moreover, the use of doping with transition metals like Co and Ni has been shown to further enhance the performance of the HER by altering the electronic structure and forming more active sites.^43^ Computational techniques, and especially density functional theory (DFT), have been fundamental in the study and design of advanced electrocatalysts. DFT can be used to understand reaction mechanisms, adsorption energies, and electronic properties, allowing rational catalyst design. Nevertheless, conventional DFT computations are computationally costly and time-consuming, which restricts their use in large-scale catalyst screening.^44^^,^^45^ The development of heterojunctions, band gap engineering, and charge separation processes has greatly enhanced photocatalytic efficiency.

Based on the literature analysis, it could be realized that a comprehensive and thorough understanding of the mechanisms and chemical kinetics of catalytic reactions based on the chalcogen group is indispensable when aiming to look for the most suitable and optimal solution in improving HER. Nonetheless, traditional models, which consume a huge amount of labor and money, are running into certain difficulties, particularly in complex catalytic systems, showing that data-driven modeling, with the support of machine learning (ML), has been emerging as a potential alternative in catalysis studies.^46^^,^^47^ It is undeniable that ML methods are becoming popular in solving complex problems in a large number of fields, such as medical, education, engineering, environment, economy, technology, and society.^48^^,^^49^^,^^50^^,^^51^^,^^52^ MLs have also become potent for overcoming the challenge and speeding up catalyst discovery.^53^^,^^54^ More importantly, combining ML with catalysis is found to enable catalyst discovery by identifying optimal band structures and reaction conditions. Recent research shows that ML models, such as random forests, support vector regression, and gradient boosting algorithms, can be used to predict with high accuracy using large datasets.^55^ For the hydrogen production process using a catalyst, ensemble ML models, trained on thousands of data points, have been found to be highly accurate in predicting HER performance metrics. These methods greatly lower computational cost and allow the screening of many candidate catalysts. Large-scale datasets have played a crucial role in promoting data-driven catalyst discovery, for example, the Open Catalyst Project (OC20) dataset, which offers comprehensive data on adsorption energies and relaxed structures for a large number of catalytic systems. These types of datasets can be used to train universal ML models that predict catalytic behavior in a wide range of materials.^56^^,^^57^ Recent research indicates that substantial gains in prediction accuracy and generalization are achieved with the use of multiple datasets and fine-tuning strategies.

Although significant advances have been made in developing catalysts for HER, major challenges remain that impede their practical implementation. Key issues include long-term stability under harsh electrochemical conditions, the scalability of synthesis routes, and cost-effectiveness for industrial applications. Moreover, a fundamental gap persists in understanding degradation mechanisms and in system-level optimization to reduce the gap between laboratory-scale performance and real-world implementation. Moreover, although ML and high-throughput computational methods have accelerated catalyst discovery, many existing works have focused more on prediction than optimization and do not consider the use of physical constraints, which results in solutions that are not necessarily practical. In this regard, this paper fills these gaps by suggesting a unified framework that integrates interpretable ML with physics-guided optimization for rational catalyst design. This work is novel because it simultaneously combines data-driven prediction, feature-level interpretability, and constraint-based optimization to guarantee physically consistent and application-relevant results. Unlike traditional methods, the given methodology focuses on the accuracy, reliability, and feasibility of the designed catalysts. The main goal is to create a scalable and robust framework that can be used for the selection of the best catalyst within realistic constraints. The main content of the paper includes the dataset and the feature engineering process, the ML modeling and interpretability analysis, the optimization framework, the main findings and comparative analysis, and directions for future research.

## Results

### Univariate feature correlation

**Figure 2:**
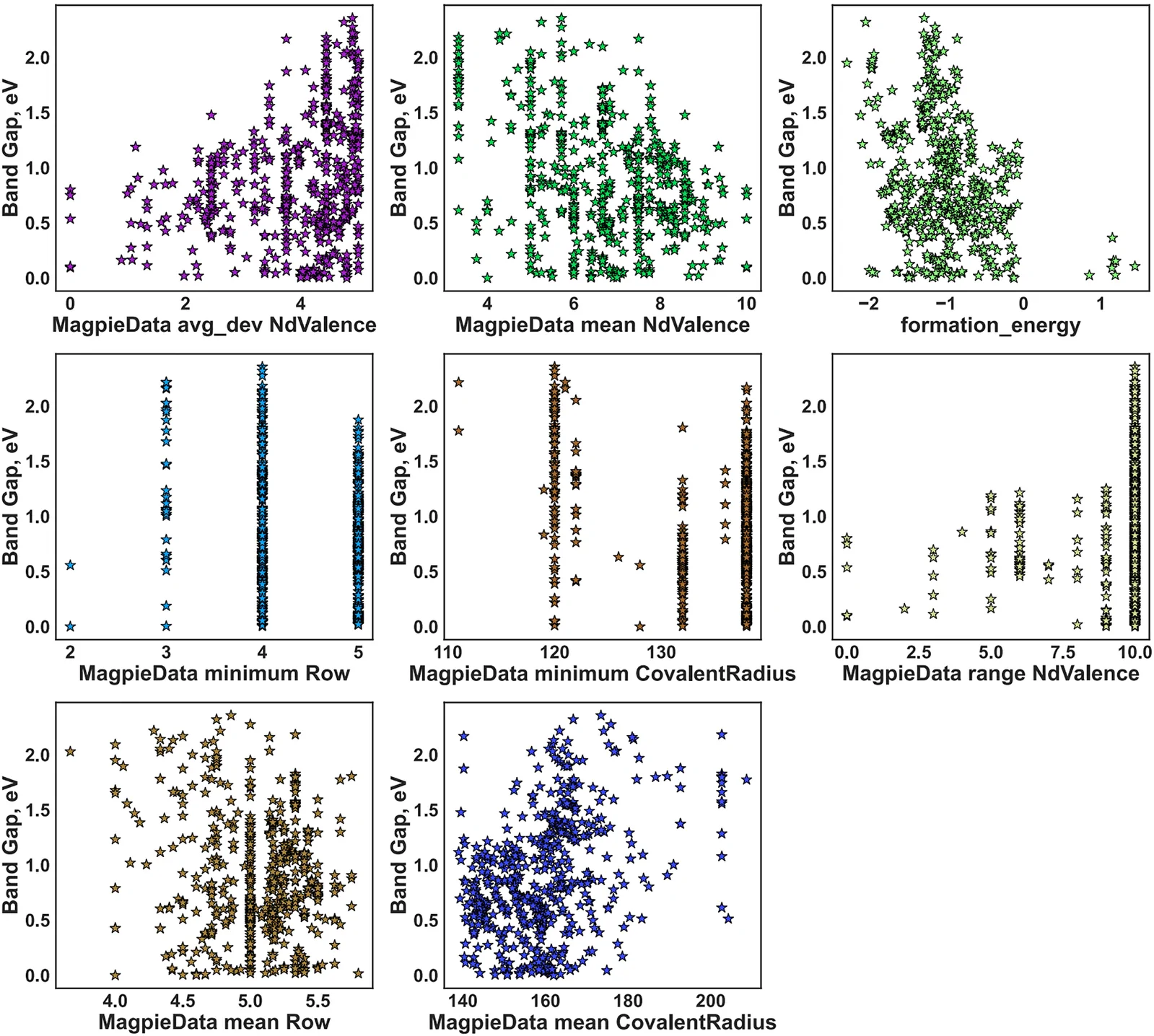
Univariate feature correlation analysis

Figure 2 represents an analysis of the relationship between features and the target variable, as univariate feature correlation or scatterplot matrix analysis. This helps identify trends, nonlinearity, clustering behavior, and potential feature importance before model interpretation.^58^

In Figure 2, different Magpie descriptors exhibit varying degrees of correlation with band gap, indicating their significance. The Magpie data average_dev Nd valence shows a moderately dispersed but slightly increasing trend, suggesting that higher variability in valence electrons may contribute to larger band gaps. In contrast, Magpie data mean Nd valence demonstrates a weak negative correlation, where increasing average valence electrons tends to reduce band gap values, possibly due to enhanced metallic character. The formation energy plot reveals a clearer inverse relationship, where more negative formation energies (indicating higher thermodynamic stability) are generally associated with larger band gaps, highlighting a key physicochemical link between stability and electronic structure. The categorical vertical distributions observed in Magpie data minimum row and minimum covalent radius indicate that these features take discrete values corresponding to elemental properties, and their dispersion suggests that specific elemental groups influence the band gap differently. Similarly, the range Nd valence shows clustering at higher values, implying that a wider distribution of valence electrons may not linearly enhance the band gap but contributes to variability. The mean row feature exhibits a dense cluster without a strong trend, indicating limited individual predictive power. Overall, the absence of strong linear relationships among most features indicates that the band gap is governed by complex, nonlinear interactions between the descriptors, justifying the use of ensemble ML models.

### Distribution analysis of target and descriptor variables

**Figure 3:**
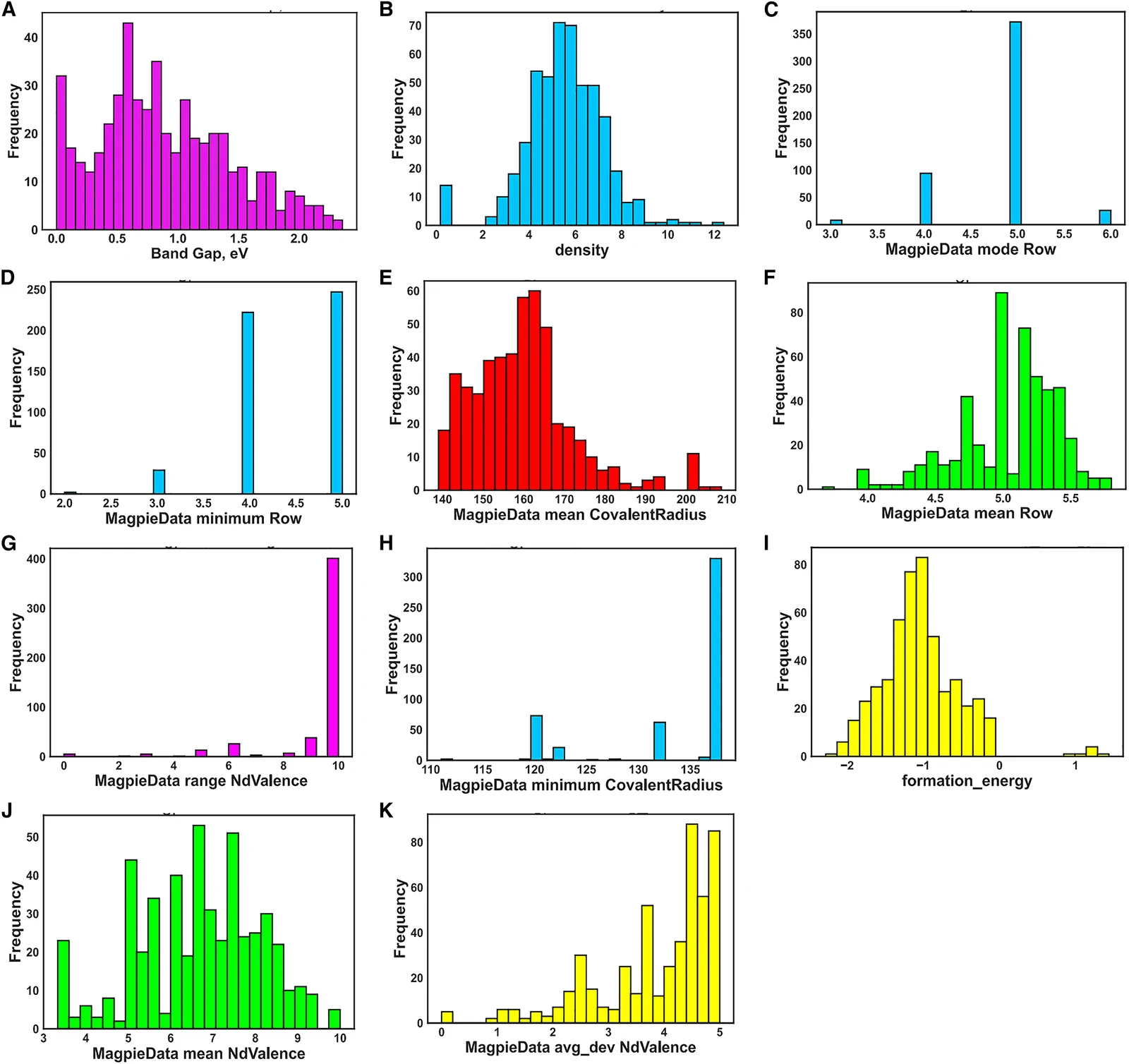
Univariate distribution analysis of the target variable and catalyst descriptors (A) Band gap distribution. (B) Density distribution. (C) Distribution of Magpie data mode row. (D) Distribution of Magpie data minimum row. (E) Distribution of Magpie data mean covalent radius. (F) Distribution of Magpie data mean row. (G) Distribution of Magpie data range Nd valence. (H) Distribution of Magpie data minimum covalent radius. (I) Formation energy distribution. (J) Distribution of Magpie data mean Nd valence. (K) Distribution of Magpie data average deviation of Nd valence.

The univariate distribution analysis of the target variable and input descriptors is shown in Figure 3. The distribution of the band gap shown in Figure 3A is moderately skewed to the right, with the majority of the data points falling in the band gap region between about 0.5 and 1.5 eV, and fewer points at higher band gap values (>2 eV). This distribution suggests that the dataset is not as skewed with the chalcogen catalyst and that there are not as many extreme cases. The density distribution exhibited in Figure 3B is essentially bell-shaped, with a mean distribution around 5–6, and is reasonably balanced without obvious bias. Moreover, Figures 3C and 3D show distributions of Magpie data mode row and Magpie data minimum row, respectively, that are categorical, with peaks at particular rows on the periodic table. In Figure 3E, the mean covalent radius distribution shows moderate fluctuation with a value of 150–170, whereas in Figure 3F, the mean row shows a fairly small fluctuation. The range Nd valence in Figure 3G is very biased with a major peak around 10, meaning that the range of valence electrons in catalysts is not very different. In a similar manner, there are peaks in the minimum covalent radius in Figure 3H, which can be linked to particular groups of elements. In Figure 3I, the formation energy is mostly negative and negatively skewed, which is consistent with the fact that most catalysts are thermodynamically stable. Distributions of mean Nd valence (Figure 3J) and avg_dev Nd valence (Figure 3K) show wider spreads, suggesting that they are capable of capturing the compositional complexity and electronic variability.

### Correlational heatmap

**Figure 4:**
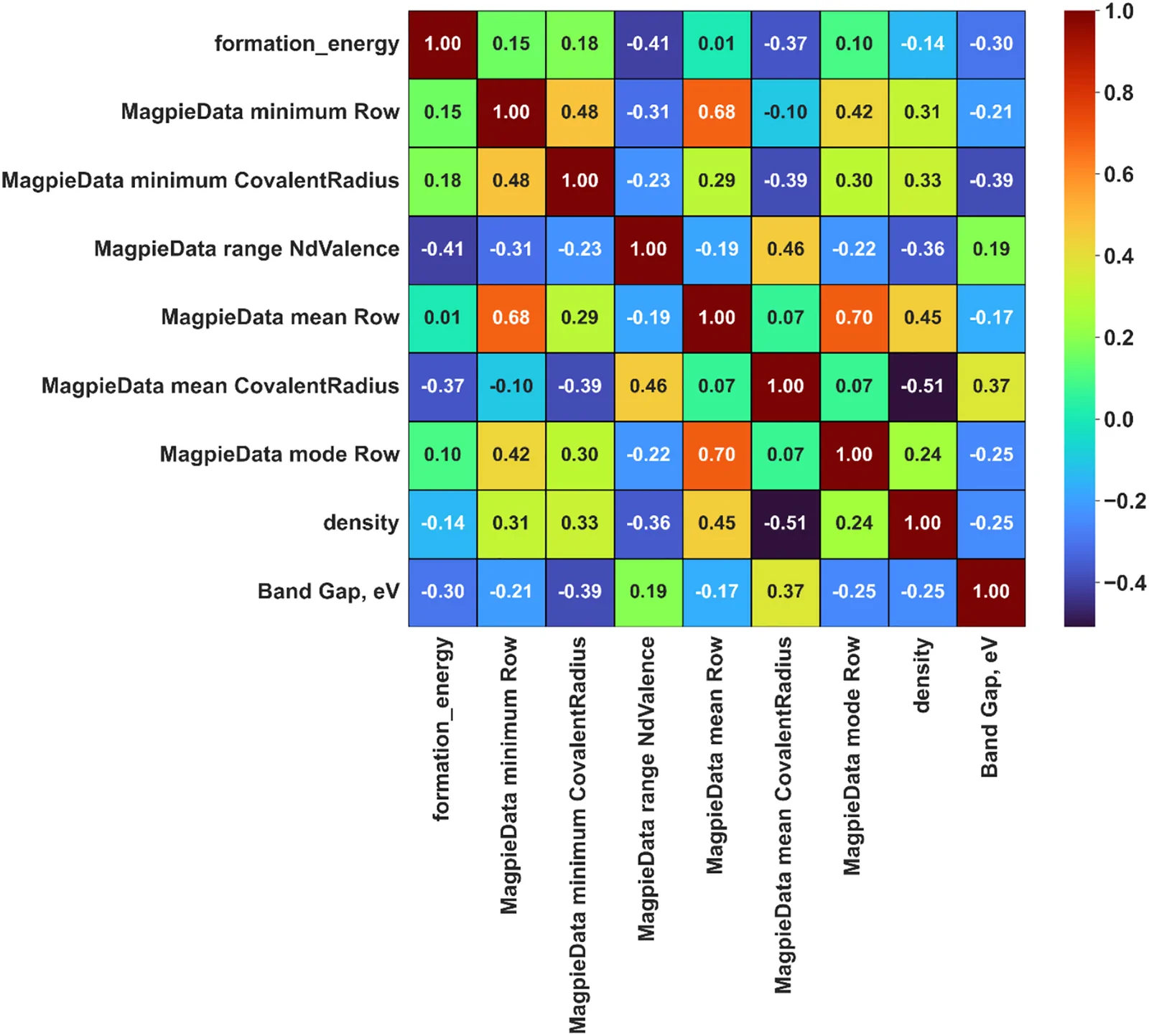
Feature correlation heatmap

Figure 4 presents the correlation heatmap analysis of input descriptors and the target variable (band gap) for chalcogen catalysts. A strong negative correlation is observed between Magpie data avg_dev Nd valence and Magpie data mean Nd valence (−0.83), indicating significant redundancy and potential multicollinearity. Similarly, Magpie data mean Nd valence shows a strong negative correlation with Magpie data mean covalent radius (−0.84), suggesting that chalcogen materials with higher valence electron averages tend to have smaller atomic radii. Among descriptors, Magpie data avg_dev Nd valence exhibits a strong positive correlation with Magpie data range Nd valence (0.72), and Magpie data mean covalent radius (0.68), reflecting consistent relationships within chalcogen compositional variability metrics.

It can be seen from Figure 4 that there is a positive correlation with Magpie data mean covalent radius (0.37) and Magpie data avg_dev Nd valence (0.30), suggesting that larger atomic size and higher valence variability slightly favor increased band gap in chalcogen catalysts. Conversely, negative correlations are observed with Magpie data minimum covalent radius (−0.39), Magpie data mean Nd valence (−0.37), formation energy (−0.30), and density (−0.25), indicating that denser, more stable, and electronically rich chalcogen systems tend to exhibit lower band gaps. Additionally, strong inter-feature correlations, such as Magpie data mean row with Magpie data mode row (0.70) and minimum row (0.68), highlight redundancy among periodic descriptors. The relatively low correlation values between most features and the band gap (|r| < 0.40) suggest that no single descriptor dominates the prediction, reinforcing the need for ensemble and nonlinear models to capture complex interactions in chalcogen catalysts.^59^

### Principal-component analysis

**Figure 5:**
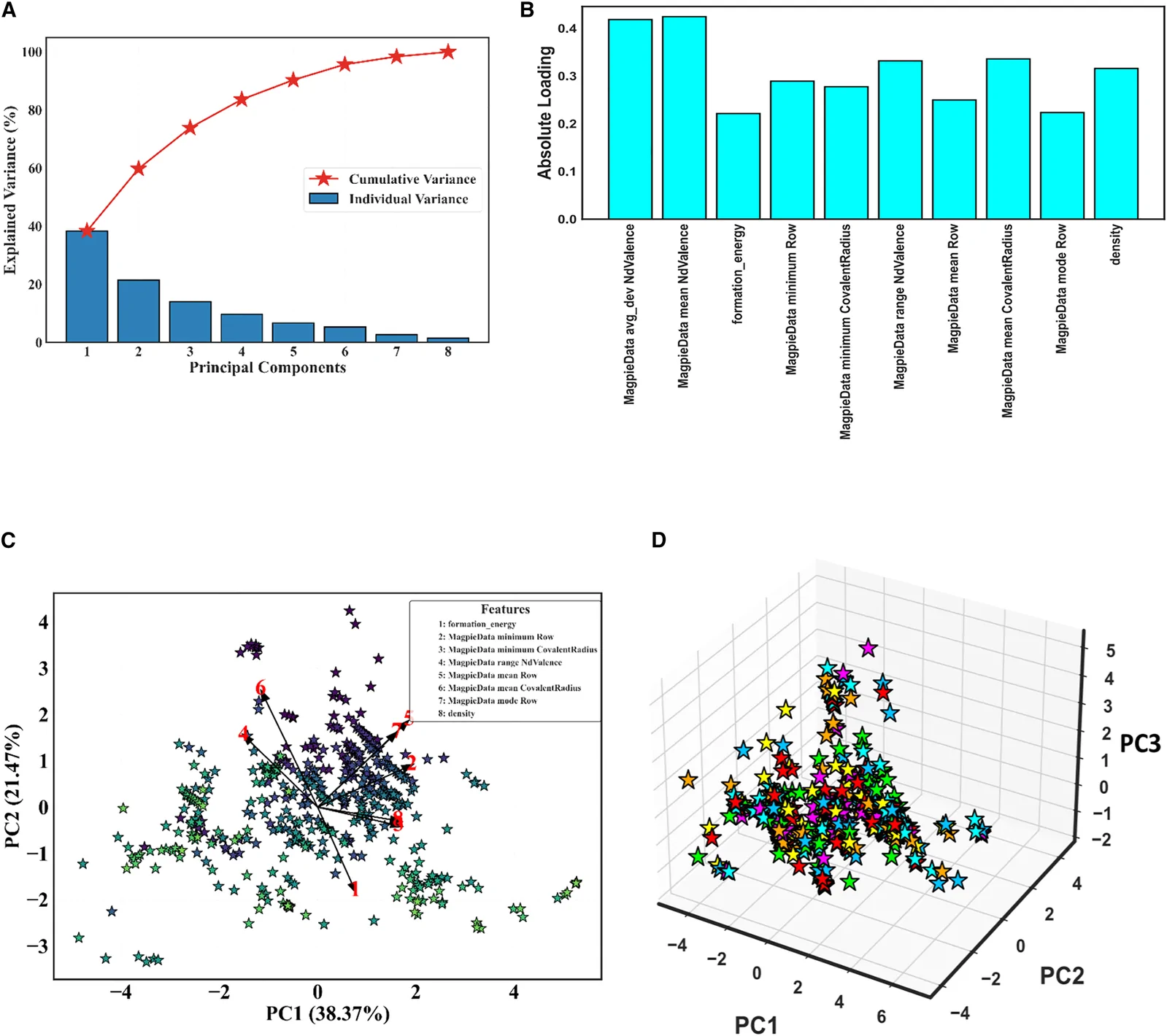
Principal-component analysis (PCA) for dimensionality reduction and feature contribution (A) Scree plot showing the explained variance. (B) PCA biplot (PC1 vs. PC2). (C) PC1 feature contributions (absolute loadings). (D) Three-dimensional PCA score plot (PC1-PC2-PC3).

Figure 5 shows principal-component analysis (PCA) to examine the variance distribution, feature importance, and inherent data structure in a lower-dimensional space. The scree plot (Figure 5A) indicates that the first principal component (PC1) captures 43.87% of the total variance, and the second principal component (PC2) captures almost 20%. The first two elements combined account for over 60% of the total variance, which means that a large part of the data information can be projected onto two dimensions. The cumulative explained variance is greater than 80% with the introduction of the third and fourth components, which validates that most of the variability in the dataset is explained by a small set of principal components. This confirms the usefulness of PCA for minimizing dimensions without compromising important information. The PCA biplot in Figure 5B is a two-dimensional representation of the data in the PC1PC2 space. The data points are widely dispersed without forming distinct clusters, and this indicates that the data lacks a high degree of inherent grouping or demarcation of classes. This observation implies that the relationship between the descriptors and target variables is not discrete. The direction and the length of the feature vectors in the biplot indicate the contribution of the feature and the direction of its influence. Such characteristics as Magpie data mean Nd valence and Magpie data avg_dev Nd valence demonstrate a high level of alignment along the PC1 axis, which means that they are the most important for explaining the main variance in the dataset. Conversely, descriptors such as Magpie data mean row and Magpie data minimum row have a greater contribution along the PC2 axis, which is indicative of their effect on secondary variations in terms of periodic trends.

The PC1 feature contribution plot (Figure 5C) also measures the impact of each descriptor with absolute loadings. Indeed, Magpie data avg_dev, Nd valence, and Magpie data mean Nd valence have the greatest contributions, with loading values of 0.42–0.43. Other characteristics like Magpie data mean covalent radius and density also have significant contributions, with values ranging from 0.31 to 0.34. In comparison, formation energy shows a relatively smaller contribution, with a loading value of nearly 0.22, meaning that it plays a less important role in defining the primary variance explained by PC1. These findings indicate that the variability of the dataset is mostly controlled by the electronic and atomic-scale descriptors. The PC1, PC2, and PC3 plot shown in Figure 5D is a three-dimensional visualization of the data that helps to obtain a more detailed picture of the data distribution. The lack of distinct clusters supports the complexity of the underlying relationships between the descriptors. In general, the PCA results show that the dataset is affected by a variety of interrelated features, and no single descriptor dominates the variance. The intermediate correlations and the distributed variance structure validate the necessity of multivariate, nonlinear ML models to effectively describe the complex interactions that determine catalyst properties such as band gap and formation energy.

### Prognostic analysis

**Figure 6:**
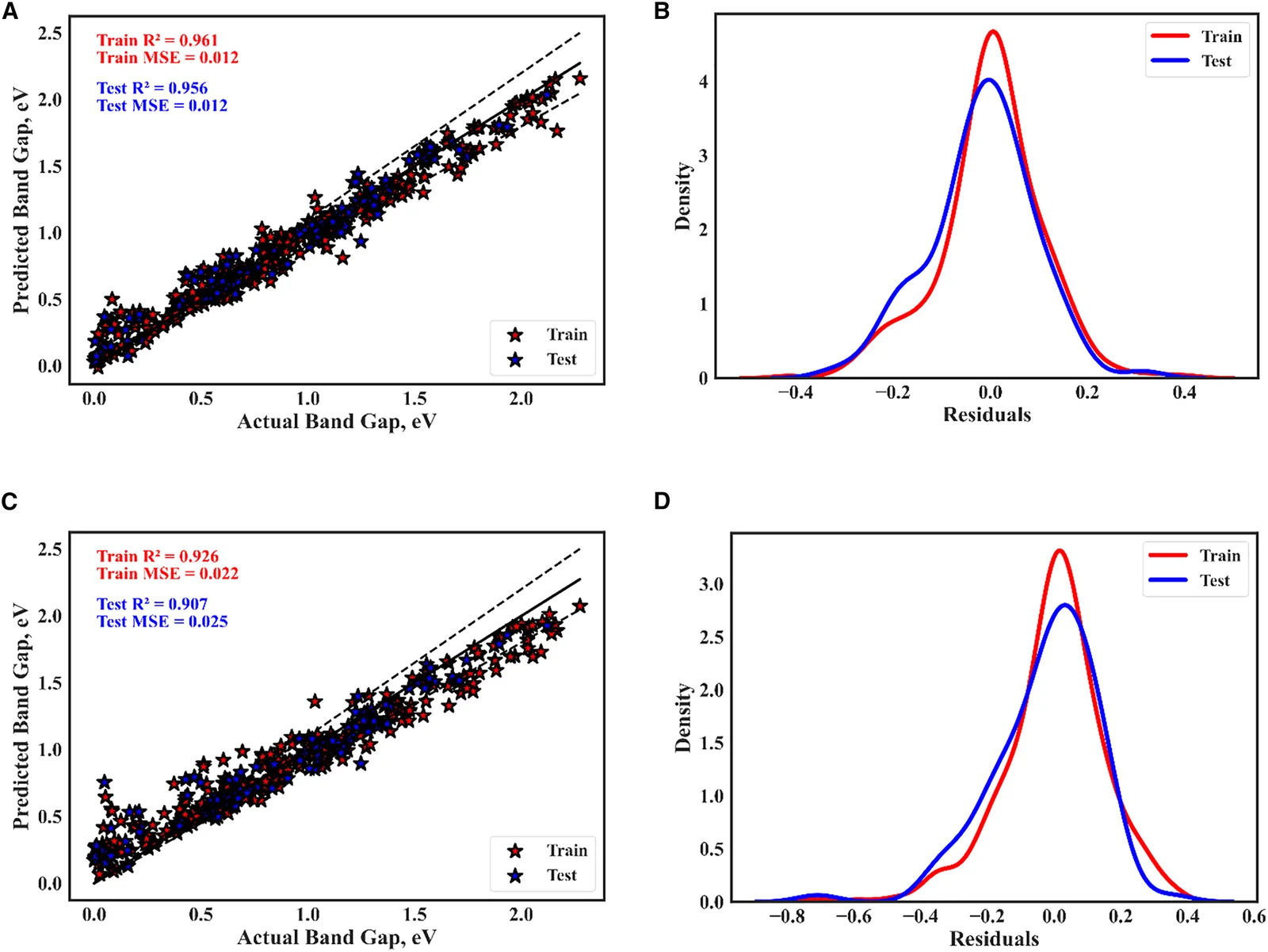
Comparative prediction performance of the machine learning models (A) Scatterplot of predicted versus experimental values for XGBoost. (B) Kernel density estimation (KDE) of prediction errors for XGBoost. (C) Scatterplot of predicted versus experimental values for random forest (RF). (D) KDE of prediction errors for RF.

Figure 6 provides a detailed comparison of the predictive performance of the XGBoost and RF models. Figure 6A indicates that the predicted and actual scatterplots of XGBoost have a high degree of predictive accuracy and consistency, with strong clustering of data points around the diagonal reference line. The model has a training performance of R^2^ of 0.961 with a low MSE of 0.012, indicating that it can effectively learn complex nonlinear relationships in complex training data. Nonetheless, the test result slightly reduces to R^2^ of 0.956 and MSE of 0.012, implying a moderate ability to generalize.^60^ These observations are further supported by the residual distribution of XGBoost in Figure 6B, where the training residuals cluster around a small and sharply peaked distribution near zero, suggesting low prediction error and high training accuracy. Notably, both distributions are symmetric about zero, meaning there is no systematic bias, and the model does not always over- or under-predict. The training hyperparameters and their optimized range are listed in Table 1.

**Table 1:** Hyperparameters, their search range, and optimized values

Model	Parameter	Search range	Value
XGBoost	Numbers of estimators	[100, 800]	799
XGBoost	Maximum depth	[3, 10]	10
XGBoost	Rate of learning	[0.01, 0.3]	0.12
XGBoost	subsample	[0.5, 1.0]	0.54
XGBoost	Col sample bytree	[0.5, 1.0]	0.87
XGBoost	γ	[0.0, 5.0]	0.04
XGBoost	Reg alpha	[0.0, 1.0]	0.19
XGBoost	Reg lambda	[0.0, 2.0]	1.78
XGBoost	Random state	Fixed	42
RF	Numbers of estimators	Fixed	500
RF	Random state	Fixed	42

Comparatively, the predictive ability of the RF model presented in Figure 6C is slightly lower. The training outcomes give an R^2^ of 0.926 and MSE of 0.022, which, though good, are inferior to those of XGBoost and suggest relatively less efficient learning of complex feature interactions. The test performance also falls to an R^2^ of 0.907 and MSE of 0.025, indicating lower generalization performance. The scatterplot indicates that the points are more dispersed and farther away from the diagonal line for band gap larger values, indicating that RF has more difficulty in capturing nonlinear relationships in more complex areas of the dataset. This weakness can be explained by RF’s averaging capability, which can even out extremely volatile forecasts and minimize sensitivity to small variations. Furthermore, the residual Kernel density estimation (KDE) plot of RF in Figure 6D shows that the training and test residuals exhibit wider distributions than with XGBoost. The greater dispersion indicates a larger prediction error and lower precision, even though it is centered at zero. Also, the test residual curve is slightly asymmetrical and with heavier tails, indicating some overestimation or underestimation in some areas.

### Model explanation with SHAP

**Figure 7:**
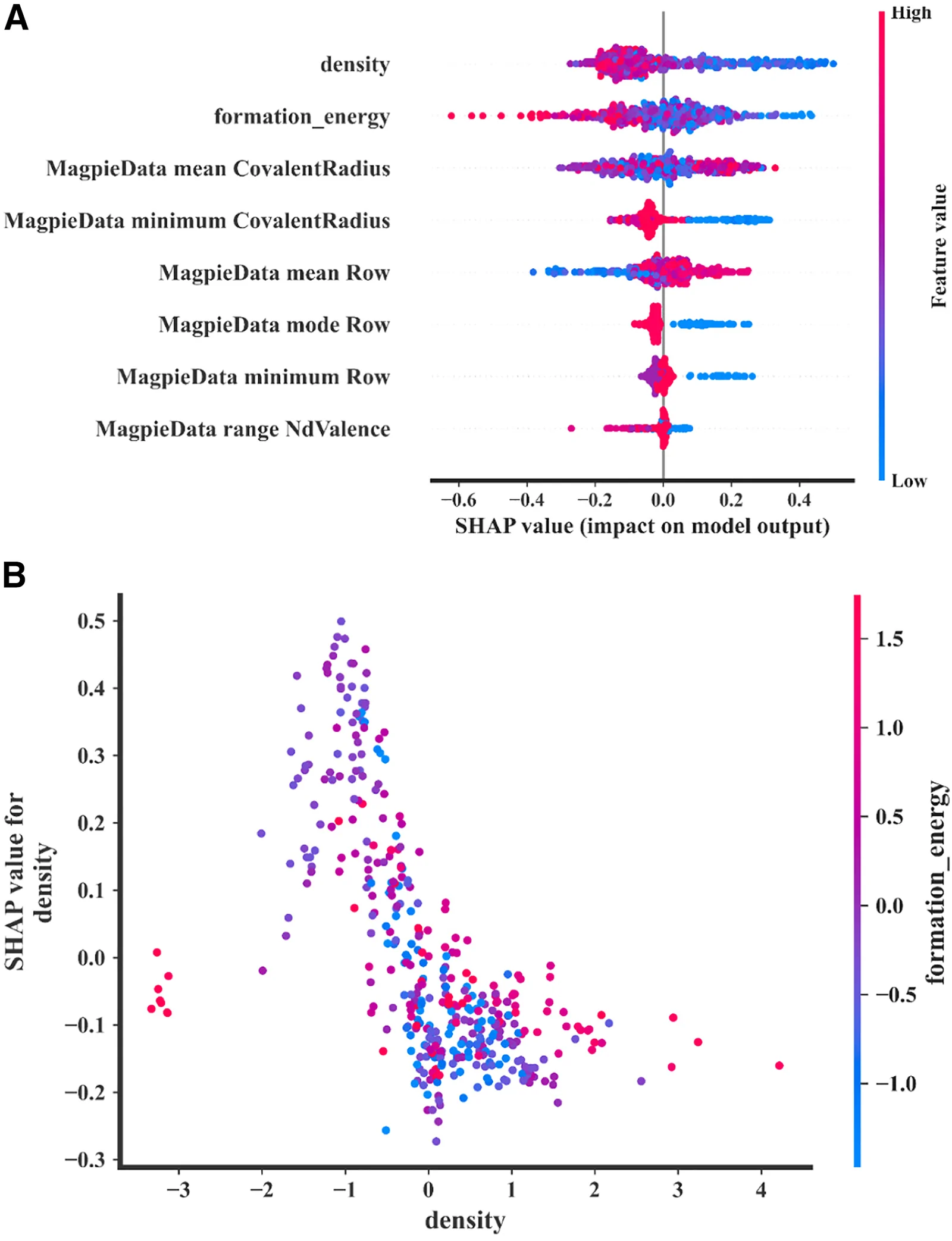
SHAP-based interpretability analysis of the XGBoost model (A) SHAP summary plot showing global feature importance and feature impacts on band gap prediction. (B) SHAP dependence plot.

The detailed explanation of the XGBoost model in SHAP (SHapley Additive exPlanations), which shows the global importance of features and the local interactions between them that determine the band gap prediction, is depicted in Figure 7. A summary plot of SHAP (Figure 7A) ranks the input features in order of overall importance in making the model predictions. The most influential descriptor is density, with values ranging approximately from −0.35 to +0.55, which means that it has a strong influence on the predicted values of the band gap. The second most important feature is the formation energy, with SHAP values ranging between approximately −0.60 and +0.45. The color gradient indicates that the lower density values (blue points) tend to increase the band gap prediction (SHAP values up to +0.5), while higher density values (red points) tend to decrease the band gap prediction (SHAP values around −0.3). Likewise, low values of formation energy correlate with positive values, and high values of formation energy correlate with negative SHAP values. The compositional descriptors that are moderately influential are Magpie data mean covalent radius and Magpie data minimum covalent radius (predominantly −0.2 to +0.3). Other features like mean row, mode row, minimum row, and range Nd valence have relatively smaller impacts, and the SHAP magnitudes are mostly less than ±0.15. The dependence plot, also shown in Figure 7B, emphasizes the nonlinear relationship between the density and the prediction of the band gap. When the density is increased from −1.5 to 0, the SHAP value drops from +0.45 to −0.25, showing that it is the low-density catalysts that strongly affect the band gap to make it larger, while it is the high-density catalysts that make it smaller.

### Multi-dimensional performance metrics and feature uncertainty

**Figure 8:**
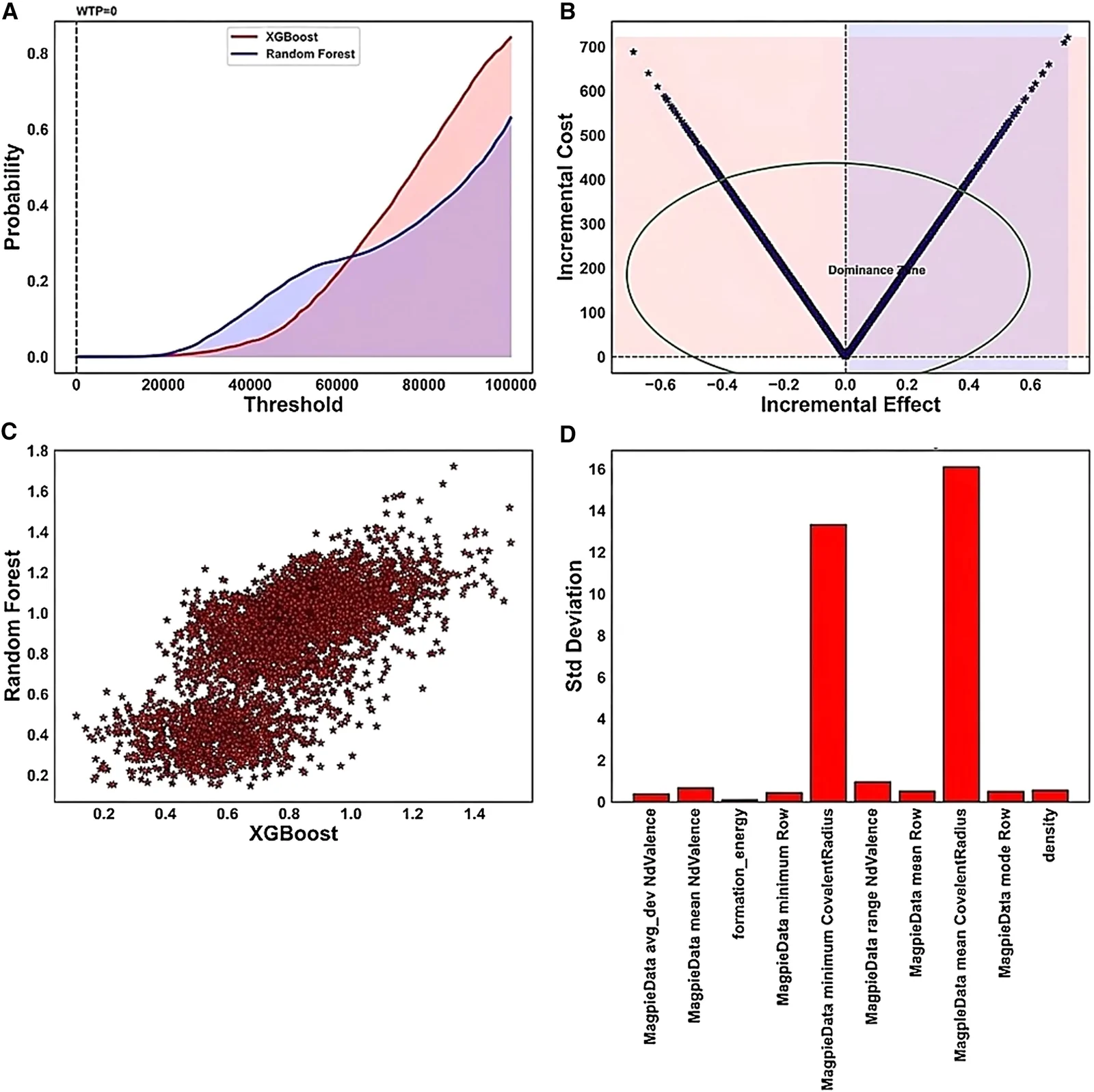
Comprehensive performance and stability analysis of the predictive models (A) Probability-based acceptability curve across varying thresholds. (B) Cost-effectiveness plane illustrating incremental shifts in effects and costs. (C) Correlation between random forest and XGBoost predictions. (D) Distribution of feature-level uncertainty based on the standard deviation of covalent radius and valence-related descriptors.

Figure 8 presents a combined comparative evaluation of the model performance, robustness, and uncertainty features of the XGBoost and RF. The probability-based acceptability curves in Figure 8A illustrate each model’s reaction to increasing threshold values. Below 30,000 thresholds, both models have probability values close to zero, which implies that they are not predictable under conservative regimes. As the threshold increases, RF initially exhibits a more rapid increase, reaching approximately 0.25 probability at about 50,000, but XGBoost lags slightly behind. However, above 60,000, XGBoost surpasses RF and increases exponentially, reaching over 0.80 at 100,000, versus about 0.60 for RF. This implies that XGBoost is more efficient when conditions are less strict and when it provides greater confidence in areas where predictions are of the most value. Figure 8B also compares the incremental behavior of the models in terms of effect and cost on the cost-effectiveness plane. Both models are V-shaped, with the center of the distribution centered on zero incremental effect and cost, although XGBoost points extend further along the positive and negative effect axes to values near ±0.7, whereas RF is a little more compact. Figure 8C shows a scatterplot of RF vs. XGBoost predictions, which has a high positive correlation with most points falling along a diagonal trend between 0.2 and 1.4. Nevertheless, there is a clear spread, especially toward higher predicted values, with XGBoost giving slightly better results than RF. This suggests that XGBoost is more likely to capture higher variance and possibly more complex nonlinear relationships, but RF is also likely to give smoother predictions. Lastly, Figure 8D shows the feature-level uncertainty using standard deviation analysis. Attributes like Magpie data mean covalent radius and minimum covalent radius are much more varied, with standard deviations of over 13 and 16, respectively, and thus their contribution to prediction uncertainty is predominant.

### Optimization framework and decision analysis

**Figure 9:**
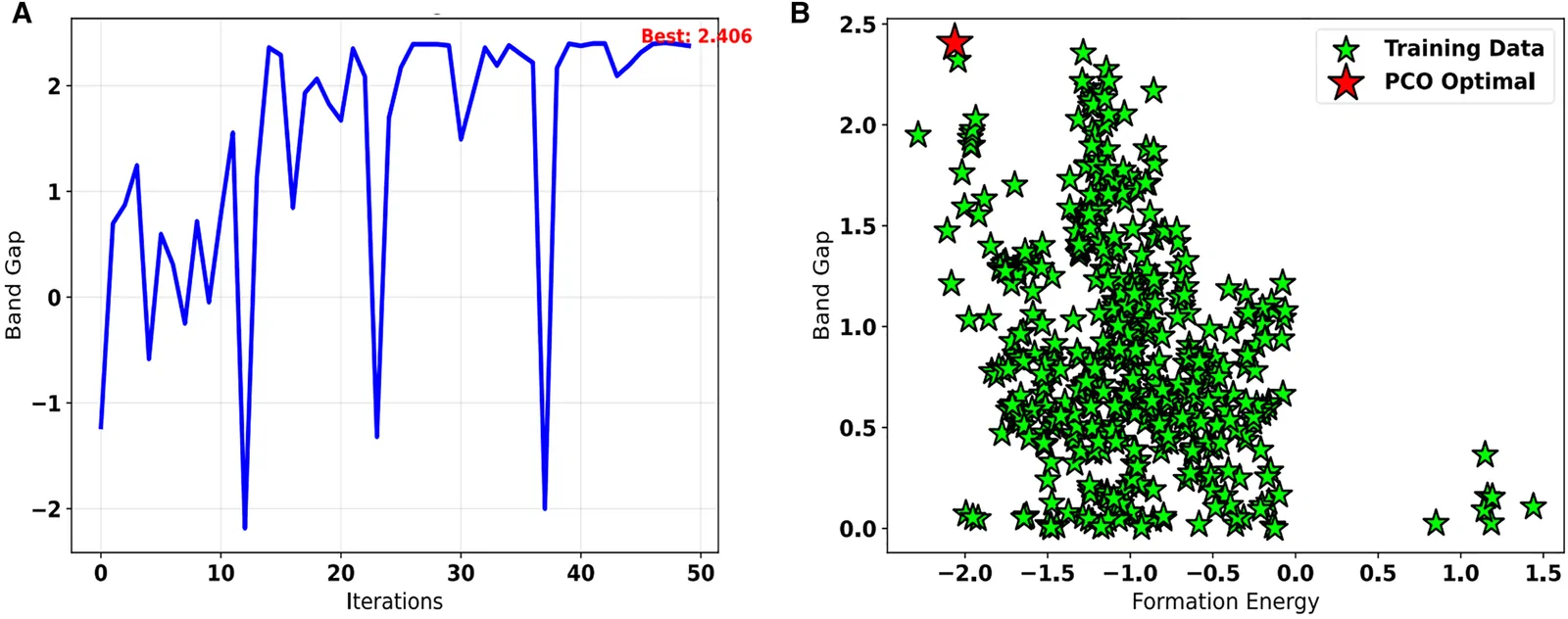
Physics-constrained optimization (A) Optimization convergence. (B) Identification of the optimal solution region.

Figure 9 gives a detailed visualization of the physics-constrained optimization (PCO) results, including convergence behavior, the location of the optimal solution, and optimal feature-level values. Figure 9A is the convergence plot indicating how the predicted band gap changes during 50 iterations, with the target value continuously improving, although some fluctuations occur due to the imposition of the penalty function. Notably, the optimization has a maximum band gap of 2.406 eV, marked in red, implying that it is effectively explored under constraints. The steep falls in iterations 12 and 37 (to values as low as −2 eV) indicate penalized solutions that do not satisfy physical constraints like positive formation energy or unrealistic density, and the constraint mechanism is indeed driving the search into feasible areas. The optimal region plots in Figure 9B show the band gap versus formation energy with the training data (lime stars) widely spread in the formation energy range between about −2.2 and 1.5 eV and the band gap between 0 and 2.3 eV. The PCO optimum (red star) is found around a formation energy of approximately −2.0 with a band gap of approximately 2.4 eV, well within the physically stable (negative formation energy) and high-performance space, illustrating the effectiveness of constraint-based optimization.

**Figure 10:**
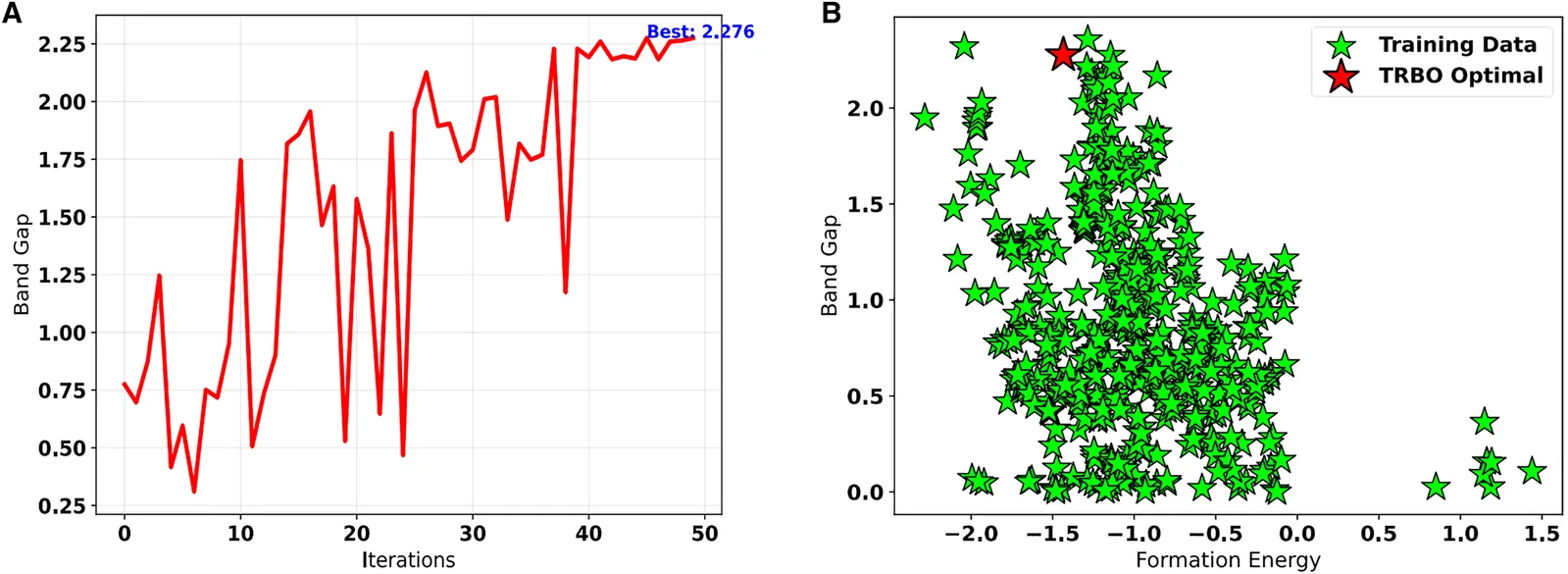
Trust-region Bayesian optimization (A) Optimization convergence. (B) Identification of the optimal solution region.

Figure 10 shows the performance of the trust-region Bayesian optimization (TRBO) model in terms of convergence behavior, a graphical representation of the optimal solution, and optimal feature values. Figure 10A shows the convergence curve, which shows a steady increase in the predicted band gap with the number of iterations, starting at approximately 0.7 eV and reaching a final value of 2.276 eV, the best value obtained, as indicated by the blue color. Compared with the PCO output, the TRBO trajectory has fewer jagged fluctuations and fewer drastic drops, which means that the exploration is stable and driven solely by the surrogate model without any applied penalty. Nonetheless, there are small swings of 0.5–2.2 eV, indicating further exploration within the trust region to strike a balance between local refinement and global search. Figure 10B presents the optimal region plot, which indicates the distribution of the training data (lime stars) between formation energy values of about −2.2 and 1.5 with band gaps of 0–2.3 eV. TRBO’s optimal solution (red star) is found around a formation energy of approximately −1.4 and a band gap of approximately 2.2 eV, which means that the optimizer can find a high-performance region. Nonetheless, the chosen point is somewhat displaced to a lower formation energy region, indicating the lack of harsh physical punishment. In general, TRBO is a good way to explore the search space to find high band-gap configurations with stable convergence behavior, but without explicit constraints, it can explore more of the search space that might be physically constrained than PCO.

### Comparative analysis of PCO and TRBO

**Figure 11:**
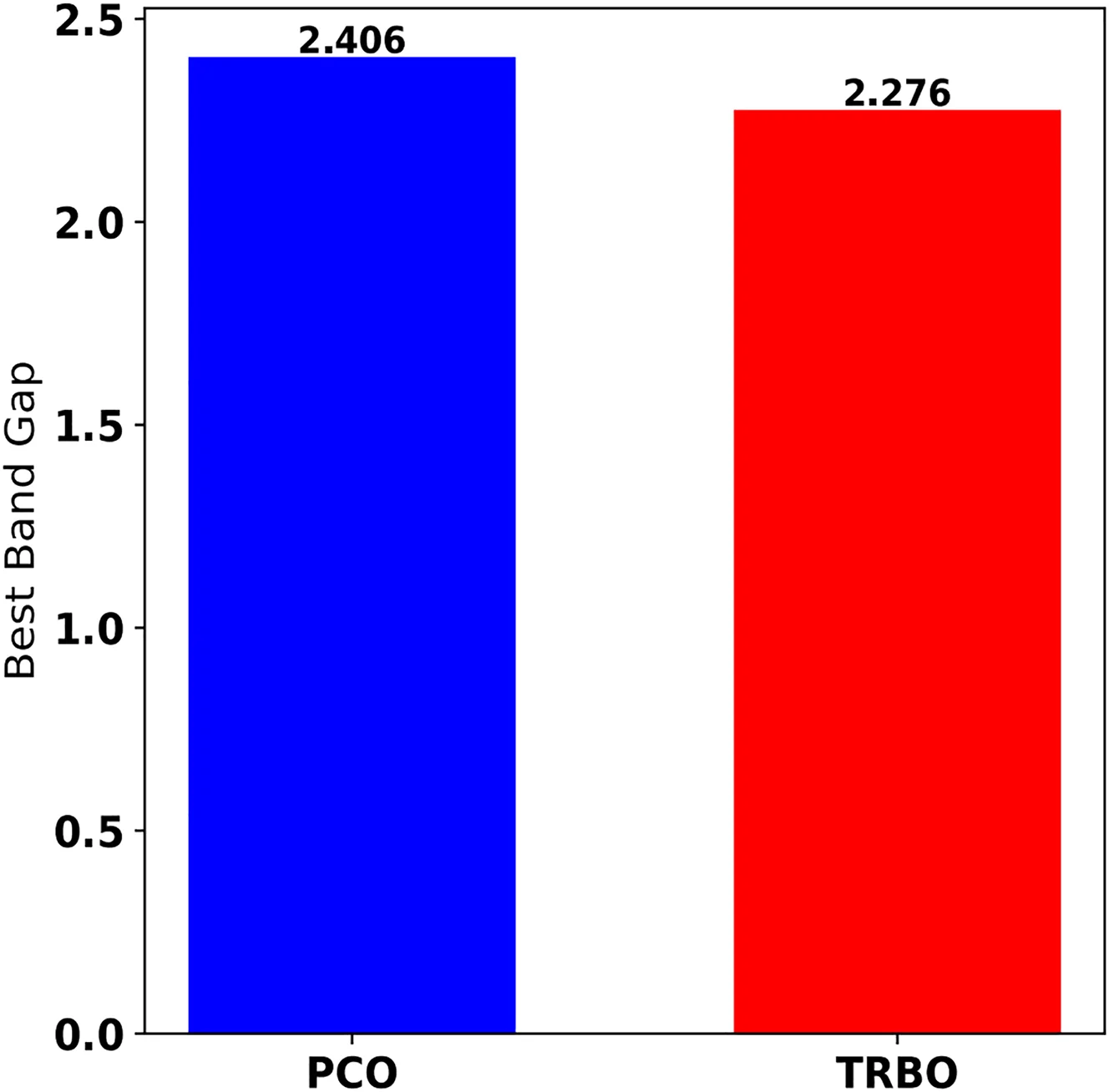
Comparison of the optimization methods

Figure 11 presents a direct comparative analysis of the optimization performance achieved using PCO and TRBO, highlighting both the quantitative outcomes and the underlying design characteristics. As shown in the bar plot, PCO achieves a superior maximum band gap of 2.406 eV, compared with 2.276 eV obtained with TRBO, indicating a clear improvement when physical constraints are incorporated into the optimization framework. This enhancement is further supported by the optimized parameter sets. For PCO, the optimal solution corresponds to a formation energy of −2.0619, ensuring strong thermodynamic stability, along with a density of 4.305 and a mean covalent radius of 208.61, which collectively contribute to improved electronic properties. Additionally, while both methods converge to similar values for the Magpie data range Nd valence (10.000001) and the mean covalent radius (around 208.66), notable differences arise in structural descriptors such as the Magpie data mean row (4.92 for PCO vs. 3.67 for TRBO) and mode row (5.36 vs. 3.00), indicating that PCO identifies a more consistent and physically meaningful descriptor space.

To make a fair comparison between PCO and TRBO, both algorithms were initialized with the same set of feasible design samples in the same parameter bounds. Furthermore, the two optimizers were given the same computational budget, consisting of the same number of initial evaluations and optimization iterations. The optimization was performed with a fixed random seed to ensure reproducibility and prevent any bias from stochastic initialization.^61^ Therefore, the variations in the final optimized band gap values can solely be ascribed to the search ability and constraint handling of the optimization algorithms, not to the initial conditions of the algorithms. The results demonstrate that although TRBO is effective at exploring the search space and identifying high-performing solutions, it cannot enforce physical realism. In contrast, PCO not only achieves a wider band gap but also ensures that the optimized catalyst configuration adheres to fundamental physical constraints, making it more reliable and practically relevant for catalyst design applications.

### Comparative performance with contemporary research

The comparative results summarized in Table 2 demonstrate that, while significant progress has been achieved in ML-based band gap prediction, most existing studies remain limited to predictive modeling without addressing the crucial aspect of optimization and physical feasibility. Gao et al.^62^ combined DFT with neural-network models to predict composition-dependent band gaps of ternary, quaternary, and quinary monolayer transition-metal chalcogenide alloys, achieving strong agreement between ML predictions and DFT results and revealing the compositional origins of band-gap bowing. Aditto et al.^63^ extended this direction from scalar properties to complete electronic band structures, demonstrating ML-based prediction of conduction- and valence-band characteristics for 2D TMD alloys while reducing the computational burden of conventional DFT calculations. Ansari et al.^64^ applied multiple linear regression to MoS_2_-based defective photonic structures and demonstrated its potential for analyzing defect-related optical responses. Kumar et al.^65^ addressed limited and imbalanced TMD/TMO datasets using oversampling and atomistic descriptors, achieving 95% classification accuracy and approximately 78% regression performance for band-gap prediction. Huang et al.^66^ further demonstrated ML-based mapping of band-gap characteristics in MoS_2_ nanotubes as a function of structural parameters. Lu et al.^67^ investigated ML-assisted MoS_2_ growth using XGBoost, SVM, NB, and MLP, while Hassan et al.^68^ combined multiple ML models with SHAP for interpretable analysis of MoS₂-based photovoltaic systems. Also, the work of Xie et al.^69^ using Crystal Graph Convolutional Neural Networks reported a band gap MAE_model of 0.388 eV compared with an MAE_DFT of 0.6 eV, indicating improved prediction over first-principles methods but without any optimization framework. Similarly, Huo et al.^70^ employed kernel-based models with RMSE values of 0.97 and 1.14 for Gaussian and linear kernels, respectively, reflecting moderate predictive capability. More advanced graph-based approaches, such as MEGNet by Chen et al.,^71^ achieved impressive MAE values as low as 0.009–0.061 eV across multiple properties, while Lv et al.^72^ reported a 30.4% improvement in prediction accuracy using a multi-feature CGCNN. In the context of band gap prediction, Wang et al.^73^ demonstrated high accuracy with R^2^ above 0.96 and MSE below 0.04 using supervised ML combined with SHAP, and Zhang et al.^74^ achieved R^2^ values greater than 0.90 with RMSE values between 0.24 and 0.47 eV across different models. Dong et al.^75^ further improved performance using deep learning and transfer learning, reporting R^2^ values greater than 90% and RMSE values around 0.1 eV. However, despite these advances, a common limitation in all these studies is the absence of a systematic optimization strategy and the lack of incorporation of physical constraints, which restricts their practical applicability in catalyst design. In contrast, the present study advances beyond prediction by integrating XGBoost with SHAP-based interpretability and a dual optimization framework comprising PCO and TRBO. While achieving strong predictive performance with a training R^2^ of 0.961 and a test R^2^ of 0.956, the key novelty lies in the ability to identify optimal catalyst configurations. The proposed PCO approach yields a maximum band gap of 2.406 eV, outperforming TRBO (2.276 eV), while simultaneously ensuring thermodynamic stability through constraints such as negative formation energy and realistic density. Unlike prior works that may achieve high accuracy but generate physically infeasible solutions, the present framework ensures improved performance and physical consistency. This integration of interpretable ML with physics-guided optimization represents a significant advancement, enabling not only accurate prediction but also reliable and practical catalyst design, thereby establishing the superiority and novelty of the proposed approach compared with existing studies.

**Table 2:** Comparative analysis of the present work with published studies

References	Chalcogen domain	ML method and optimization approach	Performance metrics
Gao et al.^62^	Monolayer transition metal chalcogenide alloys	NN + DFT (no optimization)	High agreement with DFT as well as accurate band gap prediction across compositions
Aditto et al.^63^	2D chalcogenide alloys band structure prediction	Deep learning framework	Precise prediction of full band structure
Ansari et al.^64^	MoS_2_-based chalcogen photonic structures	Multiple Linear Regression (MLR)	R^2^ > 0.90 for optical/band gap outputs
Kumar et al.^65^	Transition metal dichalcogenides and oxides	ML + oversampling	95% accuracy achieved
Huang et al.^66^	MoS_2_ nanotubes (1D chalcogen systems)	Deep learning + ML interatomic potentials	Accurate mapping of band gap vs. chirality and diameter
Lu et al.^67^	MoS_2_ growth (synthesis of 2D chalcogen catalyst)	XGBoost, SVM, NB, MLP (no optimization)	Best: MLP accuracy ∼75%, AUC ∼0.8
Hassan et al.^68^	MoS_2_-based chalcogen photovoltaic systems	Multiple MLs + SHAP	MSE: 0.47–1.23; interpretable feature importance
This work	Chalcogen-based electrocatalysts (TMD-inspired systems)	XGBoost + SHAP + PCO + TRBO	R^2^: 0.961; Test R^2^: 0.956; MSE: 0.012/0.012; Optimized Band Gap: 2.406 eV (PCO) vs. 2.276 eV (TRBO)

Eg, band gap energy (eV); Ef, formation energy (eV/atom); ρ, density (g/cm^3^); x, input feature (descriptor) vector; f(x), predicted band gap from ML model; P(x), penalty function for constraint violation; gi(x), constraint function; λ, penalty coefficient; D, design search space; Tk, trust region at iteration (k); (fˆ∗), best observed objective value; EI, expected improvement; f∗, expectation operator; R^2^, coefficient of determination; HER, hydrogen evolution reaction; MSE, mean squared error; RMSE, root mean squared error; MAE, mean absolute error; ML, machine learning; XGBoost, extreme gradient boosting algorithm; SHAP, SHapley Additive exPlanations; PCO, physics-constrained optimization; TRBO, trust-region Bayesian optimization; BO, Bayesian optimization; GP, Gaussian process; KDE, Kernel density estimation; PCA, principal-component analysis; PC1, PC2, first and second principal components.

## Discussion

The present study demonstrates that accurate prediction and optimization of the band gap in chalcogen-based catalysts require ML frameworks. These modern tools are capable of capturing complex nonlinear interactions among compositional, structural, and thermodynamic descriptors. Though preliminary statistical analysis suggested only weak-to-moderate linear relations between individual descriptors and the band gap, the combined results of the correlation analysis, PCA, and explainable ML imply that the electronic properties of chalcogen materials depend on multiple interdependent variables rather than on any single dominant one. The first principal component explained 43.87% of the total variance, and the first two principal components explained more than 60% of the total variance, indicating that the dataset was structured but highly multidimensional. Therefore, there is a need for nonlinear ensemble learning algorithms to accurately model the catalyst properties, and this explains why conventional linear approaches are not sufficient.

The algorithm with the best predictive power was XGBoost with R^2^ = 0.961 and R^2^ = 0.956 in training and testing, respectively, and MSE = 0.012 for both datasets. The close match between training and testing performance suggests excellent generalization and that the optimized model was able to avoid overfitting and capture the underlying relationships. By contrast, the RF model, with testing R^2^ = 0.907, has lower predictive power, indicating that bootstrapped aggregation is less successful in capturing local nonlinear interactions in heterogeneous catalyst datasets. One reason behind the better performance of XGBoost is its gradient boosting method, which sequentially reduces the prediction residuals and learns more and more complicated feature interactions that are hard to model by traditional bagging-based methods. These results both validate the use of gradient boosting algorithms in predicting electronic properties in chalcogen systems and echo previous studies showing the efficacy of these algorithms for materials informatics.

An essential advantage of the proposed framework is the incorporation of model interpretability, which is accomplished by SHAP analysis, turning the ML model into an interpretable scientific tool. The SHAP results showed that the most important descriptors that govern the prediction of the band gap are the density, followed by the formation energy, followed by the covalent radius and the Nd valence-related features. These observations are in line with known principles of electronic structures. This understanding is based on the fact that density directly affects interatomic spacing and orbital overlap, which in turn influence the dispersion of electronic bands, while formation energy is related to thermodynamic stability, which indirectly affects the feasibility of stable electronic configurations. Likewise, the covalent radius and the valence electron properties determine the strength of the covalent bond, the charge distribution, and the orbital hybridization, which all play a strong role in the band gap formation. Notably, the nonlinear SHAP dependence analysis also unveiled interaction effects between the two descriptors, density and formation energy, suggesting that the impact of one descriptor is contingent on the other and does not operate independently. These interactions can only be discerned by combining conventional correlation analysis with the explainable ML that underpins the understanding of the catalyst behavior.

The combination of statistical analysis and explainable ML offers valuable methodological insights, too. Although the correlation analysis did a good job of capturing feature redundancy and multicollinearity, especially within the different Magpie features, the relatively low correlation coefficients between the individual features and the target variable indicated that the band gap prediction was not well explained by pairwise linear relationships. SHAP, on the other hand, measured the actual impact of each descriptor during the learning process, which showed that some descriptors that had smaller linear relationships could still have high non-linear impacts. Thus, combining statistical feature analysis with explainable ML gives a deeper insight into the importance of the descriptors than either method can deliver and increases the level of trust in model interpretation.

This work has made a significant contribution beyond prediction; one is the integration of ML with optimization under physical constraints. Most of the previous research on band gap prediction has focused on achieving better prediction accuracy but failed to consider fundamental physicochemical requirements for optimized solutions. In the current work, both TRBO and the proposed PCO managed to find good catalyst configurations, but behaved quite differently. The maximum optimized band gap value of PCO was 2.406 eV, while TRBO was 2.276 eV (an increase of ∼5.7%). More significant is that this improvement was made at the same time; thermodynamic stability was enforced using negative formation energy and realistic density constraints. In contrast, TRBO explored the search space efficiently and showed smooth convergence behavior but lacked explicit mechanisms to prevent physically unrealistic solutions. These results illustrate that the inclusion of scientific knowledge directly in the optimization framework can improve the efficiency of the optimization and the physical reliability of the identified catalyst candidates, and limit the number of computationally optimal but experimentally impractical catalyst candidates identified. The suggested optimization scheme is of general interest to data-driven materials discovery. In high-throughput ML workflows, often the focus is just on the predictive accuracy, and not on the physical feasibility, which leads to a gap between the ML model and experimental realization. The current framework fills this gap by incorporating domain-specific constraints in the optimization procedure, and results in candidates that are both high-performance and scientifically plausible. This is especially important for catalyst informatics, which is a field where experimental synthesis is cost- and time-intensive, and physically meaningful computational screening is critical for accelerating materials development. The proposed methodology is also novel compared with previous studies. Studies in recent years have used graph neural networks, deep learning architectures, and ensemble ML for the prediction of band gaps with excellent predictive accuracy, and some studies have used SHAP to make the model less black-box. However, most of these methods are still prediction-oriented and fail to consider optimization with explicit physical constraints. In the present study, we take a step further than what has been done previously by merging accurate nonlinear prediction, interpretable feature attribution, and physics-guided optimization in a single computational framework. Thus, the proposed methodology is not just to predict the band gap value accurately but also to offer a physically feasible set of catalyst configurations, suitable for materials design and experimental validation.

In summary, this study provides a solid and understandable framework that goes beyond traditional ML prediction, combining explainable AI with PCO. This integration of XGBoost, SHAP analysis, and PCO allows for accurate prediction, mechanistic understanding, and physically realistic optimization in a single workflow. The methodology presented here offers a concrete approach to fast-track discovery and rational design of next-generation chalcogen-based catalyst materials and, more broadly, to materials informatics.

### Limitations of the study

As with most data-driven materials discovery studies, the results must be considered in the context of the dataset and the computational framework used. The proposed methodology is designed to speed the process of candidate screening and is not a substitute for detailed theoretical or experimental studies. The integrated ML and optimization framework offers explainable predictions, and the identified material configurations are candidates for further validation using more advanced computational simulations and laboratory studies. Moreover, the framework emphasizes band gap as an important screening property; inclusion of other material performance parameters in a larger multi-objective optimization framework could further aid in comprehensive electrocatalyst design for sustainable hydrogen production.

## Resource availability

### Lead contact

Requests for further information and resources should be directed to and will be fulfilled by the lead contact, Anh Tuan Hoang (tuan.hoang@ut.edu.vn).

### Materials availability

This study did not generate new unique reagents. All materials used are commercially available or previously published.

### Data and code availability

• Data reported in this paper will be shared by the lead contact upon request.
• This paper does not report original code.
• Any additional information required to reanalyze the data reported in this paper is available from the lead contact upon request.

## Acknowledgments

M.O.G.-P., E.R.-C., and A.T.H. thank the 10.13039/501100004837Spanish Ministry of Science and Innovation, project PID2021-126235OB-C32, funded by 10.13039/501100004837MCIN/10.13039/501100011033AEI/10.13039/501100011033/ and 10.13039/501100002924FEDER funds, and the financial support of Fundación UNICAJA and Erasmus+ for H2EXCELLENCE. The authors would like to thank University of Transport Ho Chi Minh city for supporting this work.

## Author contributions

D.N., conceptualization, methodology, software, validation, formal analysis, and writing – review and editing; M.O.G.-P., methodology, investigation, resources, data curation, and writing – original draft; E.R.-C., formal analysis, investigation, resources, data curation, and writing – review and editing; M.C.N., methodology, software, validation, formal analysis, investigation, resources, data curation, writing – review and editing, and visualization; H.H., formal analysis, investigation, resources, visualization, supervision, and project administration; V.H.L., formal analysis, investigation, resources, data curation, and supervision; V.H.D., data curation, writing – review and editing, visualization, and supervision; X.P.N., formal analysis, investigation, writing – review and editing, and visualization; A.T.H.: conceptualization, methodology, formal analysis, investigation, data curation, and writing – original draft.

## Declaration of interests

There is no conflict of interest associated with this publication, and there has been no significant financial support for this work that could have influenced its outcome. A.T.H. confirms that the manuscript has been read and approved for submission by all the named authors.

## Declaration of generative AI and AI-assisted technologies in the writing process

While preparing this manuscript, the authors employed ChatGPT to enhance the readability and improve the grammatical accuracy of certain sections and sentences. The use of ChatGPT is only to assist in the refinement of the text and language; we do not use ChatGPT to supplant any essential tasks.

## STAR★Methods

### Key resources table

**Table:** 

REAGENT or RESOURCE	SOURCE	IDENTIFIER
**Deposited data**		

Materials Project database	Materials Project Database	https://materialsproject.org (accessed on 26 Sep 2025)

**Software and algorithms**		

Python	Python Software Foundation	Version 3.11
MatMiner	HackingMaterials Laboratory	Version 0.9
pandas	pandas Development Team	Version 2.3.1
NumPy	NumPy Developers	Version 2.2.6
SciPy	SciPy Developers	Version 1.16.1
scikit-learn	scikit-learn Developers	Version 1.7.0
XGBoost	XGBoost Developers	Version 3.0.2
SHAP	Lundberg and Lee	Version 0.48.0
Matplotlib	Matplotlib Development Team	Version 3.10.5

### Method details

#### Data acquisition

In this study, the dataset was built by combining the Materials Project database with composition-based feature engineering techniques that were implemented in MatMiner.^76^^,^^77^ The Materials Project repository is a collection of DFT calculated catalyst properties such as formation energy, density, and electronic properties for a vast number of inorganic compounds. In this repository, a representative subset of chalcogen-containing catalysts has been curated to build predictive models for the estimation of band gaps. The chemical compositions of selected catalysts were then converted to numerical representations suitable for ML with the Magpie featurization algorithms found in MatMiner. These properties are used to describe elemental and periodic properties, like the covalent radius, the valence electron configuration, information on the row in the periodic table, and other compositional statistics, and are summarised using mathematical operators, such as the mean, minimum, range, mode, and average deviation. There were many attributes in the original descriptor space, the predictive power of which varied, so a systematic feature-selection procedure was used to identify the most informative variables. Initially, the correlation analysis was used to remove descriptors with high correlation; then, the feature-importance ranking using ensemble learning methods was used to select the most important descriptors for the target property. For this reason, eight descriptors (namely formation energy, minimum row, minimum covalent radius, Nd valence range, mean row, mean covalent radius, mode row, and density) were chosen for the input variables of the ML modeling and optimization to be carried out, and the band gap (eV) was treated as the response variable.^78^^,^^79^

#### Machine learning

##### Extreme gradient boosting

Extreme Gradient Boosting (XGBoost) builds sequential decision trees, minimizing a regularized loss via second-order gradients^80^^,^^81^:

(Equation 1) $$Obj=\sum_{i}l\left({\hat{y}}_{i},y_{i}\right)+\sum_{k}\Omega \left(f_{k}\right)\;\text{where}\;\Omega \left(f\right)=\gamma T+\frac{1}{2}\lambda ∥w{∥}^{2}$$

Each tree *f*_*k*_(*x*) predicts residuals from prior trees, with *l* of loss and Ω penalizing complexity (leaf count *T*, weights *w*). Optimal leaf scores use Taylor expansion as follows^82^:

(Equation 2) $$Obj^{\left(t\right)}\approx \sum_{i}\left[g_{i}f_{t}\left(x_{i}\right)+\frac{1}{2}h_{i}f_{t}^{2}\left(x_{i}\right)\right]+\Omega \left(f_{t}\right)$$

Where: $g_{i}=\partial l/\partial {\hat{y}}^{\left(t-1\right)}$, $h_{i}=\partial^{2}l/\partial {\left({\hat{y}}^{\left(t-1\right)}\right)}^{2}$. XGBoost handles missing data and scales to large OCP datasets.

##### Random forest

Random Forest (RF) trains *B* as an independent decision tree on bootstrapped data with random feature subsets at splits, averaging predictions^83^^,^^84^:

(Equation 3) $$\hat{y}\left(x\right)=\frac{1}{B}\sum_{b=1}^{B}T_{b}\left(x,\Theta_{b}\right)$$

Where: Θ_*b*_ denotes randomness. Reduces overfitting via bagging (variance) and feature randomness (bias).

##### SHapley additive exPlanations

SHapley Additive exPlanations (SHAP) assigns feature contributions via game theory, computing expected value shifts^85^^,^^86^:

(Equation 4) $$\phi_{i}=\sum_{S\subseteq N\setminus \left\{i\right\}}\frac{|S|!\left(|N|-|S|-1\right)!}{|N|!}\left[v\left(S\cup \left\{i\right\}\right)-v\left(S\right)\right]$$

Where: *v*(*S*) is a model output with a feature subset *S*. For tree models, TreeSHAP computes exact values efficiently. Applied to your data, SHAP reveals that covalent radius range and Nd valence drive formation energy/band gap predictions.

#### Feature engineering and descriptor selection

The concept of feature engineering is important for converting raw materials data into valuable inputs for ML models. In this work, composition-based feature extraction algorithms were used to produce descriptors, available in MatMiner, offering a full repertoire of Magpie descriptors. These descriptors describe elemental properties like atomic number, covalent radius, electronegativity, valence electron configuration, and position in the periodic table, and summarize them statistically using functions like mean, minimum, maximum, range, and mode. These representations can be used to convert complex catalyst compositions into fixed-length numerical vectors that can be trained by a model. The original feature space contained a large variety of Magpie attributes, but not all descriptors are equally predictive. Thus, a descriptor selection process was carried out to remove redundant and less informative features. Initially, correlation analysis was used to determine highly collinear variables, ensuring that multicollinearity would not negatively impact model stability. Subsequently, the most influential descriptors of the target properties, including formation energy and band gap, were determined using feature importance ranking techniques, including those based on ensemble models. This systematic selection process improves model interpretability, decreases overfitting, increases computational efficiency, and eventually leads to the creation of robust and generalizable predictive models for estimating catalyst properties.

#### Data pre-processing, model development, and training

After feature selection, the dataset underwent systematic pre-processing to ensure its robustness and consistency for model development. Missing and inconsistent entries were carefully analyzed and eliminated to ensure data integrity. Then, standardization was used to scale features and normalize the distribution of descriptors, which was expressed as:

(Equation 5) $$x^{\prime }=\frac{x-\mu }{\sigma }\text{,}$$

*μ* and *σ* denote the mean and standard deviation, respectively. This step removes bias due to variation in the magnitude of the features and enhances model convergence. The processed data were further split into training and testing datasets in an 80:20 ratio to provide a representative sample.

#### Model evaluation and performance metrics

To determine the predictive performance of the developed models, several statistical measures were used to determine accuracy, robustness, and generalizability. The coefficient of determination (*R*^2^) was used to quantify the proportion of variance explained by the model, defined as:

(Equation 6) $$R^{2}=1-\frac{\sum {\left(y_{i}-{\hat{y}}_{i}\right)}^{2}}{\sum {\left(y_{i}-\overset{¯}{y}\right)}^{2}}$$

Herein, the *y*_*i*_, ${\hat{y}}_{i}$, and $\overset{¯}{y}$ represent the actual, predicted, and mean values, respectively. Higher *R*^2^ values indicate better model performance. In addition, error-based metrics such as Root Mean Squared Error (RMSE) were employed, given by:

(Equation 7) $$MSE=\left(\frac{1}{n}\sum {\left(y_{i}-{\hat{y}}_{i}\right)}^{2}\right)$$

Training and testing datasets were used to measure model performance, assess its generalizability, and identify overfitting. Cross-validation was also done to provide consistency in results across different data splits. Moreover, the RF and Gradient Boosting Regressor.

(GBR) models were compared and evaluated to find the most predictive and reliable model. A combination of several evaluation criteria was used to guarantee a thorough and objective evaluation of the model’s performance in predicting catalyst properties.

#### Optimization framework

In this study, two novel optimization approaches, namely Physics-Constrained Optimization and Trust-Region Bayesian Optimization, were employed for the optimization of multiple parameters.

#### Physics-constrained optimization

In physics-constrained optimization (PCO), the objective is to maximize the predicted band gap while enforcing physical feasibility through penalty-based constraints. The optimization problem can be formulated as^87^^,^^88^^,^^89^:

(Equation 8) $$\underset{x}{\max}\;f\left(x\right)-\lambda \;P\left(x\right)$$

Where: *f*(**x**) represents the predicted band gap from the surrogate model, *P*(**x**) is the penalty function quantifying violation of physical constraints, and *λ* is a weighting coefficient controlling the strength of the constraint. The trade-off between the maximisation of the predicted band gap and the satisfaction of the physical constraints is controlled by the penalty coefficient λ. Small values of λ can mean that physically unrealistic solutions will remain, while very large values can mean very small solutions are the only ones that are allowed, and so the search space is too limited, and this can cause premature convergence. In this study, λ was chosen empirically based on preliminary numerical experiments to make sure that constraint violations were given sufficient penalty, but that the optimizer could explore the high-band-gap regions. The adopted value of λ has yielded good convergence behavior and ensured that the resulting candidate catalysts were physically viable, thus ensuring the balance between prediction performance and physical realism. The penalty function is defined as:

(Equation 9) $$P\left(x\right)=\sum_{i=1}^{n}\max\left(0,g_{i}\left(x\right)\right)$$

Where: *g*_*i*_(**x**) denotes constraint functions such as formation energy *E*_*f*_ > 0 or density bounds *ρ*<*ρ*_*min*_or *ρ*>*ρ*_*max*_. This formulation ensures that only physically meaningful solutions are favored during optimization.

#### Trust-region Bayesian optimization

In contrast to PCO, the Trust-region Bayesian optimization (TRBO) employs a probabilistic surrogate model and iteratively refines the search space using a trust-region mechanism. The objective is expressed as^90^^,^^91^^,^^92^:

(Equation 10) $$\underset{x\in D}{\max}\;E\left[f\left(x\right)\right]$$

Where: E[f(x)] is the expected value of the objective under a Gaussian process model. The next sampling point is selected by maximizing an acquisition function such as Expected Improvement (EI):

(Equation 11) $$\text{EI}\left(x\right)=E\left[\max\left(0,f\left(x\right)-f^{*}\right)\right]$$

**Figure 12:**
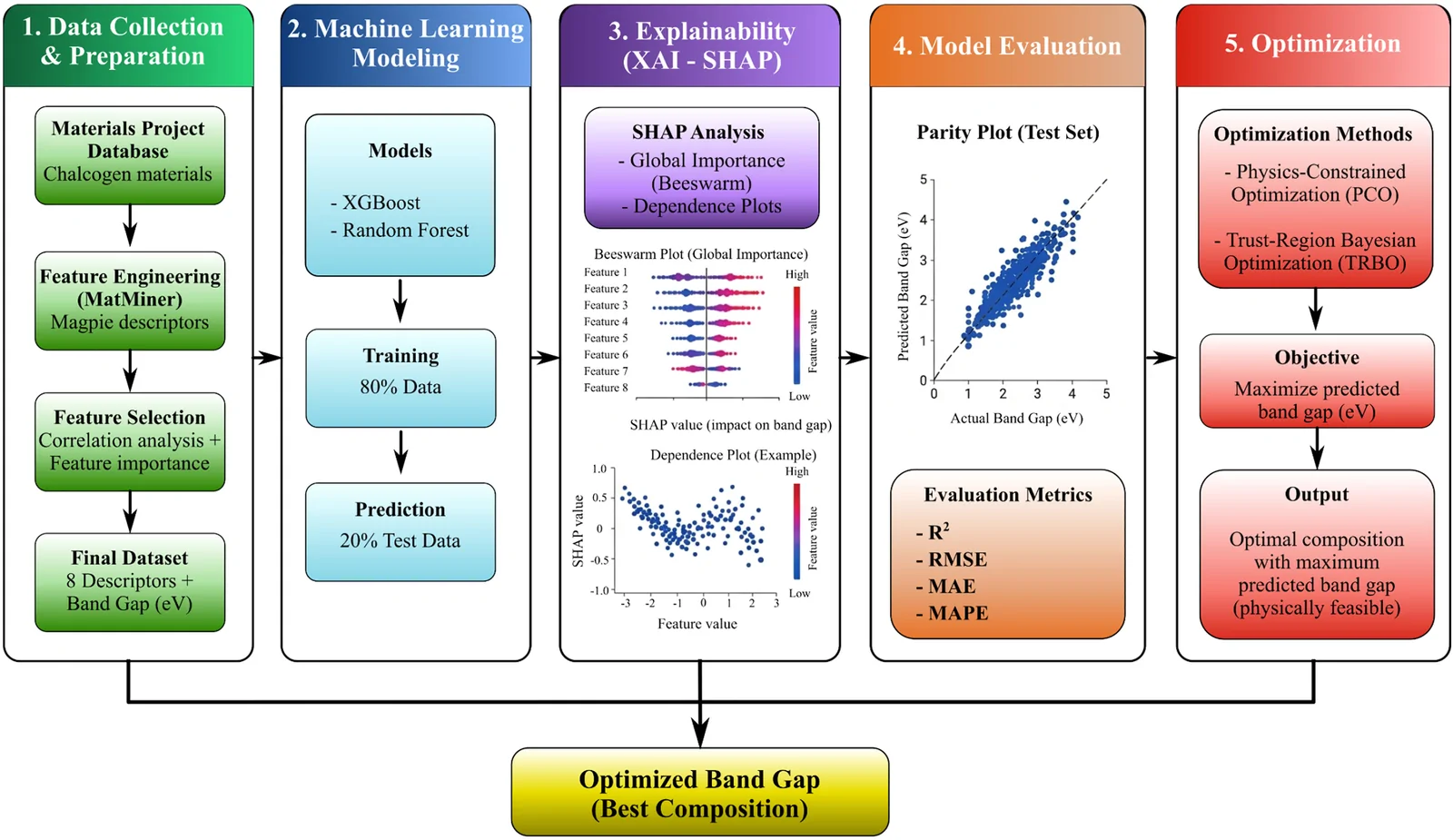
Research methodology flow chart

Where: *f*∗is the current best observed value. The search is restricted to a trust region $T_{k}\subset D$ at iteration *k*, which expands or contracts adaptively as the optimization progresses. Integrating these two approaches enables a balanced optimization strategy, in which PCO ensures adherence with physical laws through constrained maximization, while TRBO efficiently explores the design space using probabilistic inference. This dual methodology enhances both the robustness and effectiveness of band gap optimization in catalyst design. The complete research methodology used in this study is depicted in Figure 12

.
